# The optimal reconstruction size of nipple-areola complex following breast implant in breast cancer patients

**DOI:** 10.1186/s40064-016-2230-5

**Published:** 2016-05-10

**Authors:** Naomi Nagura-Inomata, Yoshiko Iwahira, Naoki Hayashi, Takako Komiya, Osamu Takahashi

**Affiliations:** Department of Breast Surgical Oncology, St. Luke’s International Hospital, 9-1 Akashi-cho, Chuo-ku, Tokyo, 104-8560 Japan; Breast Surgery Clinic, YCC Takanawa Bild., 2,3/F Takanawa, Minato-ku, Tokyo, 108-0074 Japan; Center for Clinical Epidemiology, St. Luke’s Life Science Institute, 9-1 Akashi-cho, Chuo-ku, Tokyo, 104-8560 Japan

**Keywords:** Nipple-areola reconstruction, Nipple-areola complex, Breast cancer, Skin graft

## Abstract

**Background:**

Changes in the areola size after reconstruction of the nipple-areola complex (NAC) following mastectomy and breast reconstruction with a silicon implant in primary breast cancer patients have not been well examined. This study aimed to investigate time-dependent changes in the size of the donor and graft NACs and to assess clinical factors influencing these changes.

**Methods:**

Fifty-eight consecutive patients who underwent nipple-areola reconstruction were retrospectively evaluated. Nipple-areola diameter was measured immediately after the NAC reconstruction and at each follow-up visit for at least 36 months.

**Results:**

The donor NAC constituted 81 % of the graft NAC at the time of operation. The size of the donor NAC gradually increased by up to 36.8 % after the operation. The size of the graft NAC showed a decrease by 4.5 % at 7 months, followed by recovery to the initial value. The ratio of the donor site size to the graft site size was increased at month 1 and then showed a gradual decrease to 1.08 at 36 months. A history of mastopexy or reduction for the donor site was independent factors associated with changes in the NAC size.

**Conclusions:**

To achieve symmetry, the diameter of the donor NAC immediately after the reconstruction should be at least 20 % smaller than that of the graft NAC, especially for patients without a history of additional operations.

## Background

Reconstruction of the nipple-areola complex (NAC) completes the final aesthetical step of breast reconstruction and restores the body image of breast cancer patients who have undergone mastectomy. An ideal reconstruction requires symmetry in position, size, shape, texture, and color, as well as permanent projection (Mohamed and Parodi 2011; Costa and Ferreira 2009; Nimboriboonporn and Chuthapisith 2014). NAC reconstruction is generally performed 2–3 months after the breast mound creation, as an out-patient procedure under local anesthesia. The major points of areola reconstruction are to recreate the pigmentation and texture typically associated with the opposite areola. Areola reconstruction, one of the popular procedures to achieve optimal cosmetic results, is accomplished by grafting from other sites, such as the contralateral areola or the upper inner thigh, or by intra-dermal tattooing (Farhadi et al. 2006; Bhatty and Berry 1997). Although tattooing is useful for areola reconstruction, special medical equipment, experience and periodical maintenance are required for optimize results. Therefore, grafting remains an important technique worldwide. According to Kargül et al. the best color match in NAC reconstruction was achieved by grafting from the contralateral areola rather than by grafting from the groin or tattooing (Kargül and Deutinger 2001). Although some studies have evaluated NAC reconstruction over the long term about the nipple projection (Few et al. 1999; Losken et al. 2001; Shestak et al. 2002; Banducci et al. 1999; Spear et al. 2011), long-term evaluation of the size of the areola after grafting have not been examined. In this study, we first assessed the changes in the NAC size of both donor and graft sites in patients who had undergone nipple-areola reconstruction using a full-thickness skin graft from the contralateral areola following mastectomy with breast reconstruction. In addition, we also sought to identify clinical factors influencing the size of the NAC by comparing several parameters in different groups of patients.

## Methods

### Patients

This study included 58 consecutive primary breast cancer patients who had undergone NAC reconstruction using a full-thickness skin graft from the contralateral areola following mastectomy with breast reconstruction using a tissue expander/permanent implant at the Breast Surgery Clinic from March 2006 to December 2010 and had a minimum of 3 years of follow-up (Table 1). Patients who had undergone adjuvant irradiation were excluded. This study was approved by the ethics committee of the institutional review board of St. Luke’s international hospital and permission was granted to access the patient’s data. The need for written informed consent was waived because of the retrospective nature of the study.

**Table 1:** Patient data and demographics

	Median	(Range)
Age (years)	50.0	(32–67)
Volume of the implant (cc)	310.0	(125–535)

	*n*	%
Total	58	
Age (years)		
≥50	30	51.7
<50	28	48.3
Volume of implant (cc)		
≥400	8	13.8
<400	50	86.2
Additional operations		
Mastopexy	28	48.3
Reduction	9	15.5
Augmentation	3	5.2
Timing of breast reconstruction		
Immediate	18	31.0
Delayed	40	69.0

### Reconstructive procedure

**Fig. 1:**
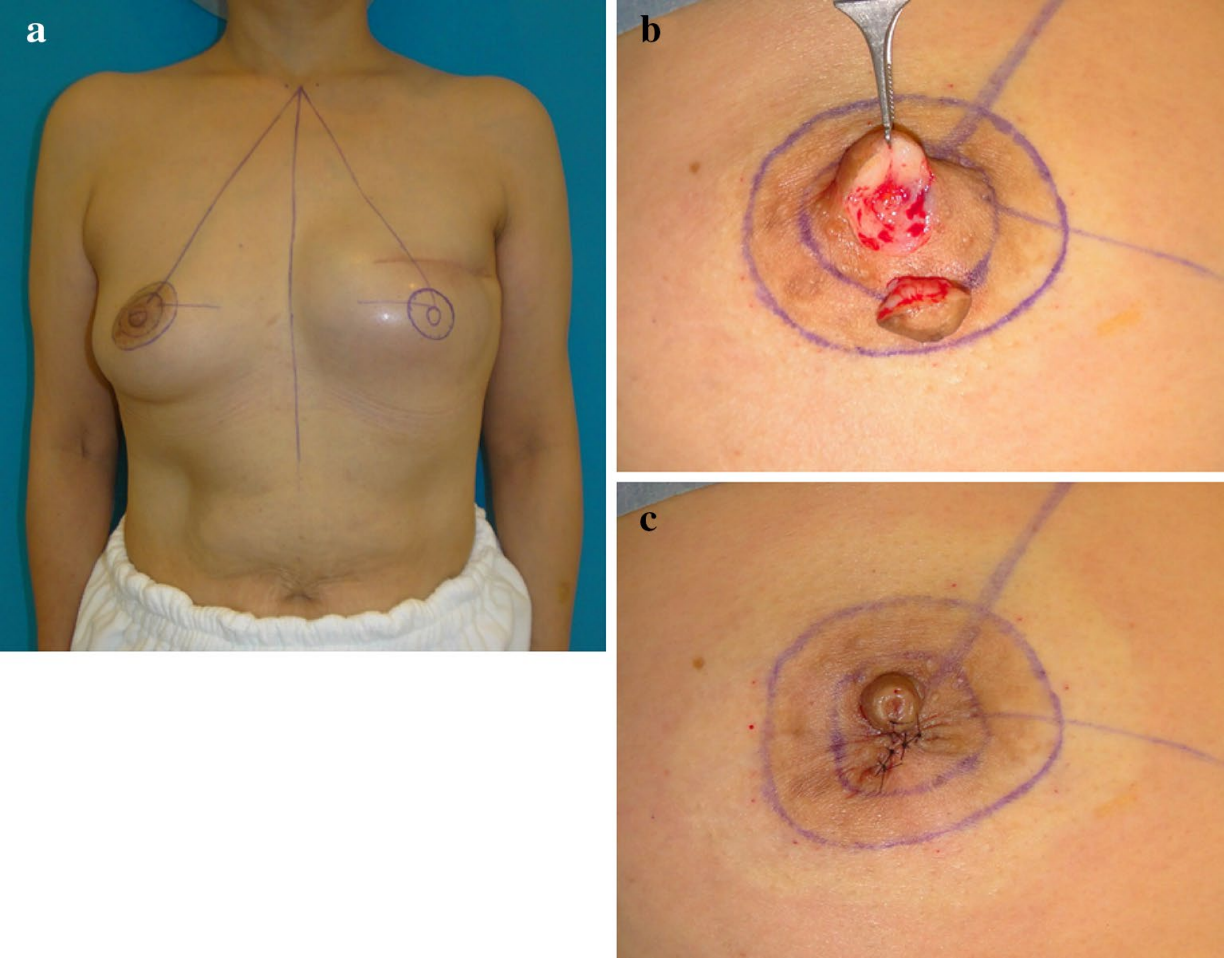
The design of nipple-areola reconstruction performed by grafting from the opposite areola. We designed the new NAC area symmetrically of the donor site. Marking to lower half of the nipple and outer rim of the areola at the donor site (**a**). A composite graft was taken from the lower half of the nipple (**b**). The original position was closed directly (**c**) at the donor site

**Fig. 2:**
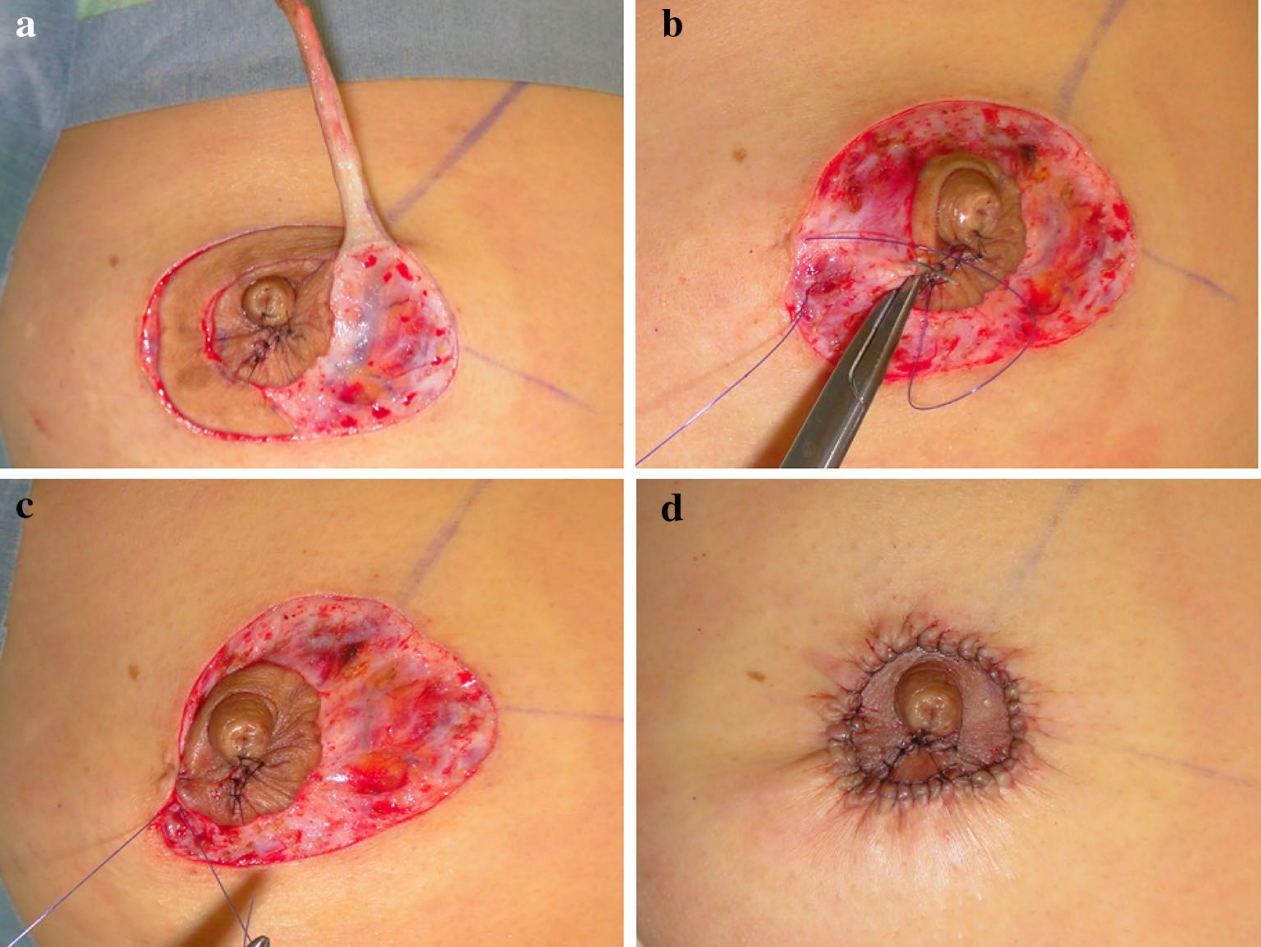
A full thickness skin graft was taken (**a**). The original location of the NAC was closed directly by suturing the superficial fascia layer at the periareolar incision (**b**, **c**). The post-operative view (**d**)

**Fig. 3:**
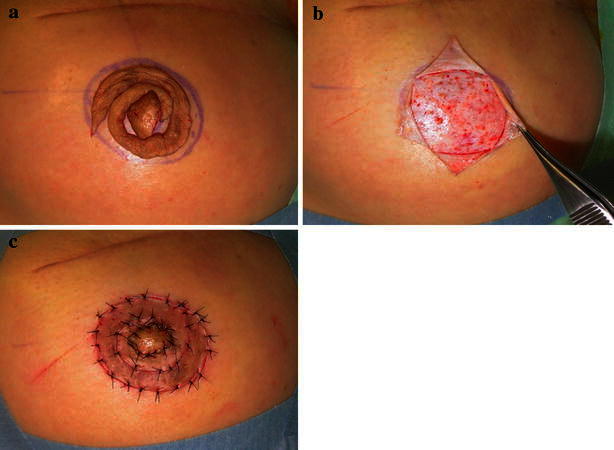
The detached skin was repositioned (**a**) and the new NAC area was de-epithelialized at the graft site (**b**). The post-operative view (**c**)

Immediate or delayed two-stage breast reconstructions were performed using tissue expanders and silicone breast implants. Nipple-areola reconstruction was performed about 3 months after the completion of the breast mound. We designed the new NAC area symmetrically of the opposite site. We marked to lower half of the nipple and outer rim of the areola at the donor site. The nipple was reconstructed using a composite graft taken from either the distal tip or the lower half of the contralateral nipple and the original position was closed directly (Fig. 1). The areola was reconstructed using a full-thickness skin graft from the outer rim of the contralateral areola, and the donor site was uniformly closed by suturing (Fig. 2). In this step, we carefully performed the suturing on the superficial fascial system, including the thick fiber bundle which located in the subcutaneous adipose tissue, to prevent the scar widening (Komiya et al. 2015). The NAC area at the graft site was de-epithelialized and the detached skin was grafted (Fig. 3). All reconstructive procedures were performed by a single plastic surgeon using the same technique. Pressure dressings were left in place for 14 days. After suture removal, the wound was supported by micropore skin tape (3 M) at the donor site to fit the wound edge for up to 6 months.

### Nipple-areola size assessment and analysis of clinical factors

**Fig. 4:**
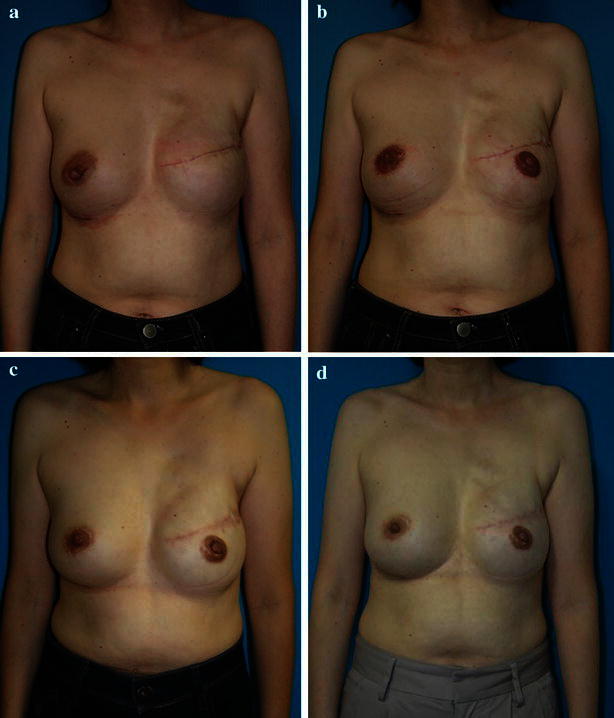
A 47-year-old woman underwent mastectomy with immediate reconstruction with tissue expander. She exchanged the tissue expander for an implant 6 months later (**a**). Four months later, a left nipple-areola reconstruction was performed by grafting from the opposite areola (**b**). The picture shows 12 (**c**) and 36 months (**d**) after the nipple-areola reconstruction

**Fig. 5:**
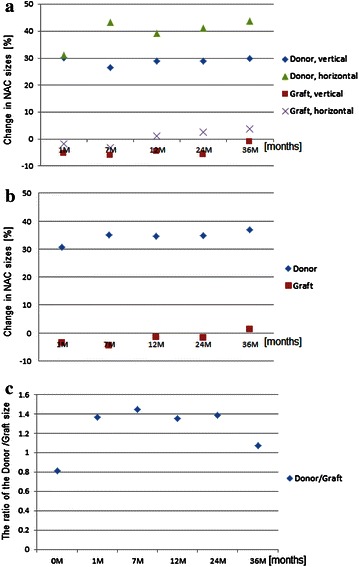
Changes in nipple-areola complex (NAC) size after reconstruction using a composite nipple graft over time, (**a**) in vertical and horizontal directions, (**b**) in the average size of vertical and horizontal directions at the donor site and the graft site, and (**c**) the ratio of donor and the graft site. The initial NAC size was counted as zero and the percentage shows the ratio of size changes

Nipple-areola diameter was measured vertically and horizontally at the time of completion (intraoperatively) and at each subsequent follow-up visit at 1, 7, 12, 24, and 36 months postoperatively (Figs. 4, 5a). The average of the two diameters (Fig. 5b) and the ratio of donor and the graft site (Fig. 5c) was calculated. The following factors were assessed for associations with changes in the NAC size: age, volume of implant, a history of additional operations such as mastopexy or reduction surgery, and timing of breast reconstruction.

### Statistical analysis

The *t* test was used for comparison of continuous variables. The multivariate linear regression analysis was performed to evaluate relationships among the variables. A value of p < 0.05 was considered to indicate statistical significance. All statistical tests were performed using SPSS version 22.0 (SPSS Inc., Chicago, IL, USA).

## Results

### Patients’ characteristics

The median age of the 58 patients was 50 years (range 32–67 years) (Table 1). The median volume of the permanent breast implant was 310.0 cc (range 125–535 cc). Thirty (51.7 %) and 28 patients (48.3 %) were aged ≥50 and <50 years, respectively. The volume of implant was ≥400 cc and <400 cc in 8 (13.8 %) and in 50 patients (86.2 %), respectively. Twenty-eight patients (48.3 %) underwent mastopexy, 9 patients (15.5 %) had a reduction surgery, and the donor site breast was augmented in 3 patients (5.2 %) before the NAC reconstruction. The breast reconstruction was performed immediately following the mastectomy in 18 patients (31.0 %), and delayed reconstruction was used in 40 patients (69.0 %).

### Time-dependent changes in the NAC size

The size of the donor NAC increased by up to 31.1 % compared to the initial size at 1 month after the reconstruction and by up to 43.7 % at 36 months in the horizontal direction. This change was larger than the change in the vertical direction (Fig. 5a). With regard to symmetry, the average diameter of the donor NAC constituted 81 % of that of the graft NAC at the time of operation. The average diameter of the donor site gradually increased by up to 36.8 % at 36 months compared to the initial value (Fig. 5b). In contrast, the size of the graft site decreased by 1.6 % at 1 month in the horizontal direction and by 5.3 % in the vertical direction (Fig. 5a). The average value gradually decreased by as much as 4.5 % compared to the initial size at 7 months and then increased by up to 1.4 % at 36 months (Fig. 5b). While the ratio of the donor NAC to the graft NAC at the operation was 0.81, this ratio increased to 1.45 at 7 months and then decreased to 1.08 at 36 months (Fig. 5c).

### Factors associated with changes in NAC size

As shown in Table 2, a history of additional operations was significantly associated with decrease in the size of the donor NAC (*p* = 0.002). The age and timing of the reconstruction did not influence the changes in the size of the donor site. With regard to the graft site, only a history of additional operations showed a trend towards influencing increase in the size of the corresponding NAC.

**Table 2:** Statistical analysis of influence factors at 36 months

	Change in size of donor sites		Change in size of graft sites	
	β	*P* value	β	*P* value
Age (years) (≥50 vs. <50)	0.002	0.56	−0.005	0.293
Volume of implant (cc) (≥400 vs. <400)	0.001	0.073	−0.0004	0.905
A history of mastopexy or reduction (yes vs. no)	−0.219	0.002	0.13	0.089
Timing of breast reconstruction (immediate vs. delayed)	0.014	0.816	−0.023	0.664

## Discussion

To the best of our knowledge, this is the first study assessing long-term changes in the NAC diameter after reconstruction using a full-thickness skin graft from the contralateral areola following mastectomy with breast reconstruction. We focus on the areola size, not the loss of nipple projection. We showed that the size of the donor NAC gradually increased for 36 months, while the size of the graft site showed only slight changes within 24 months, followed by a relatively small increase. Despite constituting the smaller size of the donor NAC, it eventually became larger than the graft NAC, especially in patients who had large implant or without additional operations.

Areola reconstruction has been achieved by grafting as well as by tattooing. Although tattooing is a useful procedure and getting popular for areola reconstruction, special medical equipment is necessary and the technique requires training and experience to optimize results. Kargul et al. stated that the best color match on NAC reconstruction was achieved by grafting from the contralateral areola rather than by grafting from the groin and tattooing (Kargül and Deutinger 2001). Moreover, patients with large areola are, rather than reconstruct the areola large by tattooing, overlooking the areola reduction surgery at the same time as the areola reconstruction. Therefore, grafting still remains an important technique worldwide.

Postoperative complications such as hypertrophy, contraction, and graft failure have been described for full-thickness grafts (Stephenson et al. 2000; Leibovitch et al. 2005). In general, however, full-thickness grafts tend to contract slightly with time, and, in this respect, our results are consistent with those of the previous studies. Interestingly, our study revealed an opposite trend for the size of the donor site. The size of the donor NAC showed and immediate increase reaching as much as 30 % one month after the operation. Importantly, the periareolar edge was sutured for both the donor and graft sites when the areola was reconstructed using a full-thickness skin graft. Therefore, the differences in NAC size dynamics are unlikely to result from variations in the surgical technique. However, it is possible that the increase in the size of the donor NAC was due to expansion of the scar in the process of wound healing. The long-term use of the micropore skin tape after suture removal to prevent later stretching of the wound likely precluded such expansion at the graft site. The tendency of the donor NAC size to increase for at least 36 months postoperatively might be related to the softness of scar caused by maturing.

Mastopexy or reduction surgery were significantly associated with reduced size of the donor site. In this regard, these surgeries resulted in additional scars around the donor areola. Moreover, hypertrophic scars were removed at the time of the NAC reconstruction. This may have contributed to the contraction of the NAC. On the other hand, large implants had a tendency to increase the size of the donor NAC, which is a reasonable explanation of the expansion of the NAC as well as the breast skin. These factors might not have affected the size of the graft site because of the small magnitude of the changes, However, According to implant size, the number of patients who received large implant was too small to confirm the association with change in NAC size in this study. Further study is warranted for this point.

The usage of the purse-string suture technique for the periareolar skin closure has been reported to reduce the expansion of the areola and the loss of nipple projection (Weinfeld et al. 2008; Caterson et al. 2015). Bodin et al. suggested that removing a part of the contralateral nipple and areola might be the most effective technique in terms of stable long-term results (Bodin et al. 2008). Although we did not assess the efficacy of these published techniques in the present study, they may be useful to prevent the expansion of the donor NAC.

Our study has some limitations. First, this study had a retrospective design. Second, the use of the adjuvant hormone therapy or changes in the body weight, that may influence the size of the breast, were not assessed in this study because of the limited amount of data. This warrants further study with a longer follow-up and larger sample size.

Our findings showed that the donor sites expanded about 36.8 % while the graft sites didn’t have a significant change. The results confirmed that the NAC size after reconstruction using a composite nipple graft was changed over time compared to the completion size. To account for this change, we should design the size of the areola at the graft site for NAC reconstruction as 20 % larger than that of donor site, to prevent the asymmetry after few years. Furthermore, it is better to re-design the size of graft site intraoperatively when suturing the donor site was done.

The judgment of the degree to enlarge the graft site should examine the individual factors of the patient. The natural processes of contraction inherent with wound healing and aging cause the change in the size of areola in all NAC reconstructions. To successfully anticipate the long-term sizes of the donor and graft NACs, graft diameter and patient factors must all be considered. The donor site is usually enlarged, but the donor site in patient with a history of additional operations does not become larger than expected. In this case, we design the graft site larger than the opposite site, but it is smaller than when designing usually.

## Conclusions

From our results, we suggest that the donor NAC should be planned at least 20 % smaller than the graft NAC during the operation considering the time-dependent changes in the NAC sizes, especially in patients without a history of additional operations.

## Abbreviation

NAC: nipple-areola complex
